# Genomic and Phenotypic Characterization of *Vibrio cholerae* Non-O1 Isolates from a US Gulf Coast Cholera Outbreak

**DOI:** 10.1371/journal.pone.0086264

**Published:** 2014-04-03

**Authors:** Bradd J. Haley, Seon Young Choi, Christopher J. Grim, Tiffiani J. Onifade, Hediye N. Cinar, Ben D. Tall, Elisa Taviani, Nur A. Hasan, AbdulShakur H. Abdullah, Laurenda Carter, Surasri N. Sahu, Mahendra H. Kothary, Arlene Chen, Ron Baker, Richard Hutchinson, Carina Blackmore, Thomas A. Cebula, Anwar Huq, Rita R. Colwell

**Affiliations:** 1 Maryland Pathogen Research Institute, University of Maryland, College Park, Maryland, United States of America; 2 CosmosID, College Park, Maryland, United States of America; 3 Food and Drug Administration, USFDA/CFSAN/DVA, Laurel, Maryland, United States of America; 4 Florida Department of Health Bureau of Environmental Public Health Medicine, Tallahassee, Florida, United States of America; 5 Florida Department of Health Bureau of Public Health Laboratories, Jacksonville, Florida, United States of America; 6 Department of Biology, Johns Hopkins University, Baltimore, Maryland, United States of America; 7 Maryland Institute for Applied Environmental Health, School of Public Health, University of Maryland, College Park, Maryland, United States of America; 8 University of Maryland Institute for Advanced Computer Studies, University of Maryland, College Park, Maryland, United States of America; 9 Bloomberg School of Public Health, Johns Hopkins University, Baltimore, Maryland, United States of America; Cornell University, United States of America

## Abstract

Between November 2010, and May 2011, eleven cases of cholera, unrelated to a concurrent outbreak on the island of Hispaniola, were recorded, and the causative agent, *Vibrio cholerae* serogroup O75, was traced to oysters harvested from Apalachicola Bay, Florida. From the 11 diagnosed cases, eight isolates of *V. cholerae* were isolated and their genomes were sequenced. Genomic analysis demonstrated the presence of a suite of mobile elements previously shown to be involved in the disease process of cholera (*ctxAB*, VPI-1 and -2, and a VSP-II like variant) and a phylogenomic analysis showed the isolates to be sister taxa to toxigenic *V. cholerae* V51 serogroup O141, a clinical strain isolated 23 years earlier. Toxigenic *V. cholerae* O75 has been repeatedly isolated from clinical cases in the southeastern United States and toxigenic *V. cholerae* O141 isolates have been isolated globally from clinical cases over several decades. Comparative genomics, phenotypic analyses, and a *Caenorhabditis elegans* model of infection for the isolates were conducted. This analysis coupled with isolation data of *V. cholerae* O75 and O141 suggests these strains may represent an underappreciated clade of cholera-causing strains responsible for significant disease burden globally.

## Introduction

*Vibrio cholerae* non-O1/non-O139 are the causative agents of sporadic, yet significant, gastrointestinal and extraintestinal infections globally, and it is well established that all strains of this species are capable of causing human infections that represent a significant global health burden [1], [2], [3], [4], [5], [6], [7]. Infection and subsequent illness caused by these organisms are linked to the presence of virulence factors in the core backbone of *V. cholerae* (hemolysins, lipases) or mobile pathogenicity islands (VPIs-1 and -2, and CTXΦ) that are frequently found in clinical isolates from cholera patients suffering severe rice water diarrhea [8], [9], [10]. Epidemic cholera is typically ascribed to *V. cholerae* serogroup O1 or O139; however, it is now understood that, similar to pathogenic *Escherichia coli*, a constellation of virulence factors along with host immune and nutritional status, are responsible for the severity and characteristic infections caused by these organisms [8], [9], [10], [11], [12]. It is established that those *V. cholerae* which acquire and express genes carried on mobile elements (O-antigens, VPI-1, VPI-2, CTXΦ, NAG-ST, etc.) are linked to epidemics of cholera. The scenario of mobile genetic element acquisition has been shown to have occurred within the 7^th^ pandemic and PG-1 and -2 clades (12), but occurrence and persistence of such genetic constellations remains underappreciated in *V. cholerae* non-O1/non-O139 (non-PG) lineages. These elements, among many others, can be laterally transferred between strains of the same species or distantly related species in the environment [13], [14], [15] and give rise to virulent strains that potentially can cause epidemics. Further, these elements can be stable in *V. cholerae* non-O1/non-O139 isolates, as in strains of the 7^th^ pandemic clade and persist in these conformations over time, ultimately conserved in the environment.

In developed nations, the leading cause of human disease caused by vibrios is consumption of raw or undercooked seafood, namely shellfish. In the United States, seafood-borne vibrioses have been traced to shellfish harvested from coastal (Atlantic and Pacific) regions, as far north as Alaska, but by far the majority of infections occur in the Gulf of Mexico, where the water temperature is warm, a parameter associated with increased *Vibrio* spp. densities as well as increased risk of vibriosis [16], [17], [18], [19], [20]. Recent cases of cholera traced to seafood consumption, and many *V. parahaemolyticus* infections and deaths caused by *V. vulnificus* have been reported in this region.

*V. cholerae* O75 serogroup strains have been reported to cause sporadic shellfish-borne cholera cases in the southeastern United States [21], [22]. Outbreaks caused by these strains are not continuous as outbreaks in developing nations because sanitation in the United States is such that untreated human waste is not typically discharged into water used for drinking, recreation, or harvesting of seafood and water used for consumption or for household use is typically treated to remove bacterial pathogens. Further, *V. cholerae* O75 strains have been isolated from environmental waters in the southeastern United States in the absence of reported cholera cases [21]. Here we present results of analysis of eight clinically recovered *V. cholerae* O75 isolates from an indigenous US Gulf Coast cholera outbreak that occurred in, 2010, and during March and April, 2011 [22].

## Materials and Methods

Clinical *V. cholerae* isolates that were epidemiologically linked to consumption of oysters harvested from the Apalachicola Bay, FL were obtained from the Florida Department of Health Bureau of Public Health Laboratories in Jacksonville, FL. The genomes described in this study were either obtained from the NCBI Genbank database or, in the case of strains CP1110, 1111, 1112, 1113, 1114, 1115, 1116 and 1117, were sequenced using the Genome Analyzer IIx system (Illumina, Inc., San Diego, CA) according to the manufacturer's methods. Raw reads of these genomes were assembled with CLC Genomics Workbench. Genome-to-genome comparisons, identification and characterization of molecular genetic elements (MGEs), as well as core genome phylogenetics were performed by using methods described previously [12]. Genomes of *V. cholerae* strains CP1110 to CP1117 were annotated using Rapid Annotation using Subsystem Technology [23]. For *in silico* genomic island BLASTN and phylogenetic analyses the RAST-annotated ORFs of *V. cholerae* CP1110 were used as a reference. PCR analyses of virulence factors not resolved by genome sequencing (*rstR* alleles, *nanH*, and *ctxB* biotype) were done using the methods of Choi et al. [24], Vora et al. [25], and Nusrin et al. [26]. Phenotypic assays (proteolysis, hemolysis, biofilm formation, and motility) were conducted following methods standardized for *V. cholerae* [27]. Hemolysis, biofilm formation, motility, and proteolysis assays were done in nine replicates. BiOLOG phenotypic microarrays (PM1, PM2A, PM9, and PM10) were conducted in duplicate following the manufacturer's instructions (BiOLOG, Hayward, CA). Substrate metabolism was scored by dividing the area under the curve by the background values. Scores >2 were considered positive for metabolism of that substrate.

For the *Caenorhabditis elegans* model, SS104 *glp-4 (bn2)* temperature sensitive sterile strain was acquired from the *Caenorhabditis* Genetics Center (CGC). SS104 worms were maintained at 16°C, and experiments were performed at 25°C. Worms were cultured in *C. elegans* habitation media (CeHM) in tissue culture flasks on a platform shaker [28]. Adult nematodes were bleached (0.5 M NaOH, 1% Hypochlorite) to collect eggs, which were incubated in M9 media for 24 hours to bring them to synchronized L1 stage, and then transferred to CeHM. L4 stage worms were transferred to assay plates for survival experiments. Pathogen lawns for survival assays along with control bacteria *E. coli* OP50 were prepared by inoculating Nematode Growth Medium (NGM), in 6-cm Petri dishes, with 50 µl of an overnight *V. cholerae* culture. Plates were incubated overnight at room temperature before worms were added. Temperature sensitive sterile worms (SS104 *glp-4(bn2)*) strain, obtained from *Caenorhabditis* Genetics Center were transferred to NGM plates containing *V. cholerae* wild type strains E7946, CP1112, CP1114, CP1115 or *E. coli* OP 50 bacterial lawns and incubated at 25°C with ∼20–30 L4 stage worms added to each plate. Animals were scored every 24 h for survival. Animals were considered dead when they no longer responded to a gentle prod with a platinum wire. *C. elegans* survival was plotted using Kaplan-Meier survival curves and analyzed by log rank test using GraphPad Prism (GraphPad Software, Inc., La Jolla, CA). Survival curves resulting in *p* values of <0.05 relative to control were considered significantly different [29]. Strains and genomes used in this study are listed in Table 1.

**Table 1:** Genomes/strains used in this study.

Organism	Strain ID	Serogroup/Serotype	Biotype	Geographical origin	Source of isolation	Year of isolation	Accession nos.
*Vibrio cholerae*	NCTC 8457	O1	El Tor	Saudi Arabia	Clinical	1910	NZ_AAWD01000000
*Vibrio cholerae*	M66-2	O1	El Tor	Makassar, Indonesia	Clinical	1937	NC_012578/NC_012580
*Vibrio cholerae*	MAK757	O1	El Tor	Sulawesi, Indonesia (Celebes Islands)	Clinical	1937	NZ_AAUS00000000
*Vibrio cholerae*	O395	O1	Classical	India	Clinical	1965	NC_009456/NC_009457
*Vibrio cholerae*	V52	O37		Sudan	Clinical	1968	NZ_AAKJ02000000
*Vibrio cholerae*	N16961	O1	El Tor	Bangladesh	Clinical	1975	NC_002505/NC_002506
*Vibrio cholerae*	E7946	O1	El Tor	Bahrain	Clinical	1978	Not Sequenced
*Vibrio cholerae*	2740-80	O1	El Tor	Gulf Coast, USA	Water	1980	NZ_AAUT01000000
*Vibrio cholerae*	TM 11079-80	O1	El Tor	Brazil	Sewage	1980	NZ_ACHW00000000
*Vibrio cholerae*	CT 5369-93	O1	El Tor	Brazil	Sewage	1980	NZ_ADAL00000000
*Vibrio cholerae*	TMA21	non-O1/non-O139		Brazil	Water	1982	NZ_ACHY00000000
*Vibrio cholerae*	12129(1)	O1	El Tor	Australia	Water	1985	NZ_ACFQ00000000
*Vibrio cholerae*	RC9	O1	El Tor	Kenya	Clinical	1985	NZ_ACHX00000000
*Vibrio cholerae*	BX 330286	O1	El Tor	Australia	Water	1986	NZ_ACIA00000000
*Vibrio cholerae*	V51	O141		USA	Clinical	1987	NZ_AAKI02000000
*Vibrio cholerae*	RC27	O1	Classical	Indonesia	Clinical	1991	NZ_ADAI00000000
*Vibrio cholerae*	INDRE 91/1	O1	El Tor	Mexico	Clinical	1991	NZ_ADAK00000000
*Vibrio cholerae*	C6706	O1	El Tor	Peru	Clinical	1991	NZ_AHGQ00000000
*Vibrio cholerae*	CP1032(5)	O1	El Tor	Mexico	Clinical	1991	NZ_ALDA00000000
*Vibrio cholerae*	MO10	O139		Madras,India	Clinical	1992	NZ_AAKF00000000
*Vibrio cholerae*	Amazonia	O1	Amazonia	Amazonas, Brazil	Clinical	1992	NZ_AFSV00000000
*Vibrio cholerae*	MJ-1236	O1	El Tor	Matlab, Bangladesh	Clinical	1994	NC_012668/NC_012667
*Vibrio cholerae*	IEC224	O1	El Tor	Belém, Brazil	Clinical	1994	NC_016944/NC_016945
*Vibrio cholerae*	1587	O12		Lima, Peru	Clinical	1994	NZ_AAUR01000000
*Vibrio cholerae*	RC385	O135		Chesapeake Bay, USA	Plankton	1998	NZ_AAKH02000000
*Vibrio cholerae*	CP1033(6)	O1	El Tor	Mexico	Clinical	2000	NZ_AJRL00000000
*Vibrio cholerae*	AM-19226	O39		Bangladesh	Clinical	2001	NZ_AATY01000000
*Vibrio cholerae*	MZO-3	O37		Bangladesh	Clinical	2001	NZ_AAUU01000000
*Vibrio cholerae*	MZO-2	O14		Bangladesh	Clinical	2001	NZ_AAWF01000000
*Vibrio cholerae*	623-39	non-O1/non-O139		Bangladesh	Water	2002	NZ_AAWG00000000
*Vibrio cholerae*	CIRS101	O1	altered El Tor	Dhaka, Bangladesh	Clinical	2002	NZ_ACVW00000000
*Vibrio cholerae*	CP1038	O1	El Tor	Zimbabwe	Clinical	2003	NZ_ALDC00000000
*Vibrio cholerae*	B33	O1	El Tor	Beira, Mozambique	Clinical	2004	NZ_ACHZ00000000
*Vibrio cholerae*	CP1040(13)	O1	El Tor	Zambia	Clinical	2004	NZ_ALDD00000000
*Vibrio cholerae*	CP1041(14)	O1	El Tor	Zambia	Clinical	2004	NZ_ALDE00000000
*Vibrio cholerae*	VC35	O1	El Tor	Kedah, Malaysia	Clinical	2004	NZ_AMBR00000000
*Vibrio cholerae*	3500-05	O1	El Tor	India	Clinical	2005	NZ_AHGL00000000
*Vibrio cholerae*	3582-05	O1	El Tor	Pakistan	Clinical	2005	NZ_AHGP00000000
*Vibrio cholerae*	3546-06	O1	El Tor	India	Clinical	2006	NZ_AHGM00000000
*Vibrio cholerae*	LMA3984-4	O1	El Tor	Belém, Brazil	River Water	2007	CP002555/CP002556
*Vibrio cholerae*	3554-08	O1	El Tor	Nepal	Clinical	2008	NZ_AHGN00000000
*Vibrio cholerae*	3569-08	O1	El Tor	Gulf Coast, USA	Environmental	2008	NZ_AHGO00000000
*Vibrio cholerae*	2009V-1046	O1	El Tor	Pakistan	Clinical	2009	NZ_AHFX01000000
*Vibrio cholerae*	2009V-1085	O1	El Tor	Sri Lanka/India	Clinical	2009	NZ_AHFY00000000
*Vibrio cholerae*	2009V-1096	O1	El Tor	India	Clinical	2009	NZ_AHFZ00000000
*Vibrio cholerae*	2009V-1116	O1	El Tor	Pakistan	Clinical	2009	NZ_AHGA00000000
*Vibrio cholerae*	2009V-1131	O1	El Tor	India	Clinical	2009	NZ_AHGB00000000
*Vibrio cholerae*	2010V-1014	O1	El Tor	Pakistan	Clinical	2009	NZ_AHGG00000000
*Vibrio cholerae*	2011EL-1137	O1	El Tor	Zimbabwe	Clinical	2009	NZ_AHGJ00000000
*Vibrio cholerae*	CP1048(21)	O1	El Tor	Bangladesh	Clinical	2010	NZ_ALDJ00000000
*Vibrio cholerae*	CP1050(23)	O1	El Tor	Bangladesh	Clinical	2010	NZ_ALDK00000000
*Vibrio cholerae*	EL1786	O1	El Tor	Haiti	Clinical	2010	NC_016445/NC_016446
*Vibrio cholerae*	EL1798	O1	El Tor	Haiti	Clinical	2010	NZ_AELI00000000
*Vibrio cholerae*	EL1792	O1	El Tor	Haiti	Clinical	2010	NZ_AELJ00000000
*Vibrio cholerae*	HE-09	non-O1/non-O139		Haiti	Environmental	2010	NZ_AFOP00000000
*Vibrio cholerae*	HE-39	non-O1/non-O139		Haiti	Environmental	2010	NZ_AFOQ00000000
*Vibrio cholerae*	HE-48	non-O1/non-O139		Haiti	Environmental	2010	NZ_AFOR00000000
*Vibrio cholerae*	HC-02A1	non-O1/non-O139		Haiti	Clinical	2010	NZ_AFOT00000000
*Vibrio cholerae*	HC-21A1	O1	El Tor	Saint-Marc, Haiti	Clinical	2010	NZ_AGUK00000000
*Vibrio cholerae*	HC-22A1	O1	El Tor	Saint-Marc, Haiti	Clinical	2010	NZ_AGUL00000000
*Vibrio cholerae*	HC-32A1	O1	El Tor	Port-au-Prince, Haiti	Clinical	2010	NZ_AGUO00000000
*Vibrio cholerae*	HC-72A2	O1	El Tor	Arcahaie, Haiti	Clinical	2010	NZ_AGUY00000000
*Vibrio cholerae*	HC-80A1	O1	El Tor	Port-au-Prince, Haiti	Clinical	2010	NZ_AGVB00000000
*Vibrio cholerae*	2010EL-1961	O1	El Tor	Haiti	Clinical	2010	NZ_AHGD00000000
*Vibrio cholerae*	2010EL-2010H	O1	El Tor	Haiti	Clinical	2010	NZ_AHGE00000000
*Vibrio cholerae*	2010EL-2010N	O1	El Tor	Haiti	Clinical	2010	NZ_AHGF00000000
*Vibrio cholerae*	2011EL-1089	O1	El Tor	Haiti	Clinical	2010	NZ_AHGH00000000
*Vibrio cholerae*	HC-41B1	non-O1/non-O139		Haiti	Clinical	2010	NZ_AJRP00000000
*Vibrio cholerae*	HE-40	non-O1/non-O139		Haiti	Hospital Latrine	2010	NZ_AJRX00000000
*Vibrio cholerae*	HE-46	non-O1/non-O139		Haiti	Gray Water	2010	NZ_AJRY00000000
*Vibrio cholerae*	HC-44C1	non-O1/non-O139		Haiti	Clinical	2010	NZ_AJSK00000000
*Vibrio cholerae*	HC-39A1	non-O1/non-O139		Haiti	Clinical	2010	NZ_ALDM00000000
*Vibrio cholerae*	HC-41A1	non-O1/non-O139		Haiti	Clinical	2010	NZ_ALDN00000000
*Vibrio cholerae*	HC-42A1	non-O1/non-O139		Haiti	Clinical	2010	NZ_ALDO00000000
*Vibrio cholerae*	HC-47A1	non-O1/non-O139		Haiti	Clinical	2010	NZ_ALDR00000000
*Vibrio cholerae*	HE-16	non-O1/non-O139		Haiti	Gray Water	2010	NZ_ALEB00000000
*Vibrio cholerae*	HE-25	non-O1/non-O139		Haiti	Gray Water	2010	NZ_ALEC00000000
*Vibrio cholerae*	HE-45	non-O1/non-O139		Haiti	Gray Water	2010	NZ_ALED00000000
*Vibrio cholerae*	CP1042(15)	O1	El Tor	Thailand	Clinical	2010	NZ_ALDF00000000
*Vibrio cholerae*	BJGO1	non-O1/non-O139		Mississippi Gulf Coast, USA	Clinical	2010	NZ_AFOU00000000
*Vibrio cholerae*	2010EL-1749	O1	El Tor	western Africa	Clinical	2010	NZ_AHGC00000000
*Vibrio cholerae*	2011EL-1133	O1	El Tor	Haiti	Clinical	2011	NZ_AHGI00000000
*Vibrio cholerae*	2011V-1021	O1	El Tor	Dominican Republic	Clinical	2011	NZ_AHGK00000000
*Vibrio cholerae*	2011EL-301	non-O1/non-O139		Taganrog, Russia	Water	2011	NZ_AJFN00000000
*Vibrio cholerae*	CP1110	O75		Florida Gulf Coast, USA	Clinical	2010–2011	NZ_AMWF00000000
*Vibrio cholerae*	CP1111	O75		Florida Gulf Coast, USA	Clinical	2010–2011	NZ_AMWS00000000
*Vibrio cholerae*	CP1112	O75		Florida Gulf Coast, USA	Clinical	2010–2011	NZ_AMWT00000000
*Vibrio cholerae*	CP1113	O75		Florida Gulf Coast, USA	Clinical	2010–2011	NZ_AMWU00000000
*Vibrio cholerae*	CP1114	O75		Florida Gulf Coast, USA	Clinical	2010–2011	NZ_AMWV00000000
*Vibrio cholerae*	CP1115	O75		Florida Gulf Coast, USA	Clinical	2010–2011	NZ_AMWR00000000
*Vibrio cholerae*	CP1116	O75		Florida Gulf Coast, USA	Clinical	2010–2011	NZ_ANNM00000000
*Vibrio cholerae*	CP1117	O75		Florida Gulf Coast, USA	Clinical	2010–2011	NZ_AMWW00000000
*Vibrio cholerae*	VL426	non-O1/non-O139	albensis	Maidstone, Kent, UK	Water		NZ_ACHV00000000
*Vibrio anguillarum*	96F	O1		Chesapeake Bay, USA	Striped bass (*Morone saxatilis*)		NZ_AEZA00000000
*Vibrio anguillarum*	RV22	O2β		Atlantic coast of Spain	Turbot (*Scophthalmus maximus*)		NZ_AEZB00000000
*Vibrio anguillarum*	775	O1		coast of Washington state, USA	Coho salmon (*Oncorhynchus kisutch*)		NC_015633/NC_015637
*Vibrio coralliilyticus*	ATCC BAA-450			Zanzibar, Tanzania	Coral	1999	NZ_ACZN00000000
*Vibrio mimicus*	SX-4			China	Clinical	2009	NZ_ADOO00000000
*Vibrio mimicus*	VM573			United States	Clinical	1990s	NZ_ACYV00000000
*Vibrio mimicus*	MB-451			Matlab, Bangladesh	Clinical		NZ_ADAF00000000
*Vibrio orientalis*	CIP 102891			Yellow sea, China	Water		NZ_ACZV00000000
*Vibrio parahaemolyticus*	RIMD 2210633	O3:K6		Japan	Clinical	1996	NC_004603/NC_004605
*Vibrio splendidus*	12B01			Plum Island Estuary, Massachusetts, USA	Water		NZ_AAMR00000000
*Vibrio vulnificus*	YJ016		biotype 1	Taiwan	Clinical		NC_005139/NC_005140
*Vibrio* sp. RC341	RC341	O153		Chesapeake Bay, USA	Water	1998	NZ_ACZT00000000
*Grimontia hollisae*	CIP 101886			Maryland, USA	Clinical		NZ_ADAQ00000000

## Results and Discussion

### Phylogenomic Analysis of Florida Outbreak Strains

**Figure 1:**
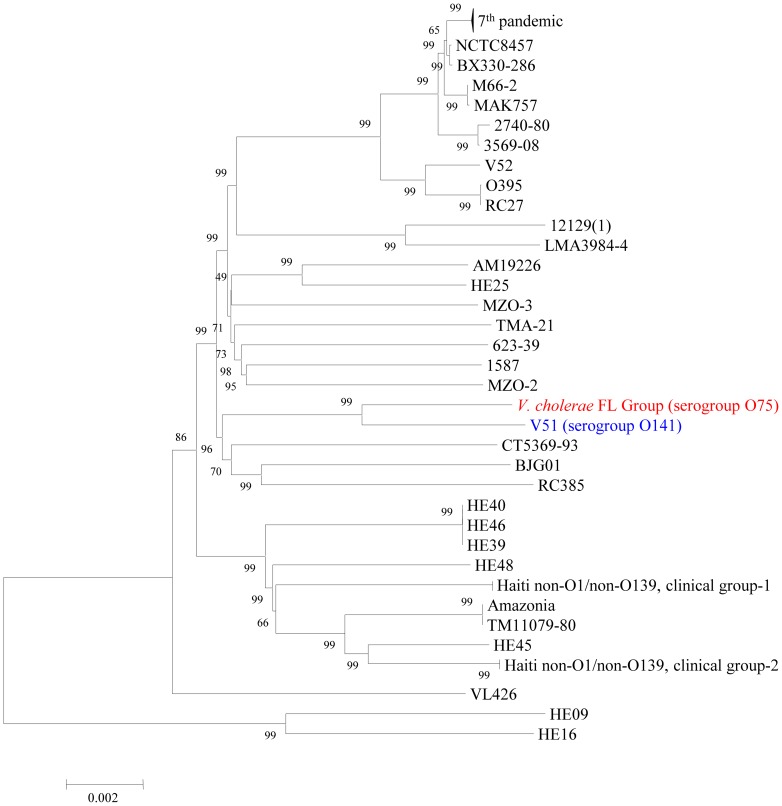
Neighbour-joining tree inferring phylogenetic relationships of 84 *V. cholerae* genomes based on 995 orthologous protein-coding genes (954,646 bp). *V. cholerae* FL Group is labelled in red and *V. cholerae* V51 is labelled in blue. Haiti non-O1/non-O139 clinical groups-1 and -2 are further defined by Hasan et al. [6]. Numbers at nodes represent bootstrap values. Nucleotide substitution model is the Kimura-2-parameter. Bar length = 0.002 nucleotide substitutions per site.

The eight isolates subjected to analysis in this study have been labeled by number (isolates CP1110, 1111, 1112, 1113, 1114, 1115, 1116 and 1117) and are hereafter collectively referred to as the *V. cholerae* FL Group. The phylogeny of 84 fully and partially sequenced *V. cholerae* strains, including the eight *V. cholerae* FL Group genomes, was inferred (Figure 1). Results of the analysis demonstrate that the *V. cholerae* FL Group are sister taxa with *V. cholerae* V51, a clinical *V. cholerae* O141 serogroup strain isolated from a human clinical case in the United States in 1987, suggesting a common ancestor after it had diverged from other *V. cholerae* lineages. From a public health perspective, the results of the analysis demonstrate the group represents a phyletic lineage of *V. cholerae* non-O1/non-O139 strains that persist in the United States as a cause of morbidity. Although, not added to this analysis due to the absence of their sequenced genomes, results of this analysis coupled with *V. cholerae* isolation data from cholera patients worldwide demonstrate that other *V. cholerae* serogroup O141 and O75 strains result in similar clinical manifestations as the strains in this study, that is symptoms of cholera [30], [31]. As with the isolates sequenced in this analysis, other *V. cholerae* O141 and O75 infections in the United States were associated with either seafood consumption or presence of the patient in a coastal state, suggesting infections with strains of these serogroups are transmitted to people in a similar manner as those of the O1 serogroup and therefore they have a similar ecology as serogroup O1 strains in the United States [32], [33].

We identified 8 single nucleotide polymorphisms (SNPs) among the *V. cholerae* O75 genomes in this study. Six of these occurred in six separate ORFs and two occurred in one ORF annotated as a “putative transcriptional activator ToxR.” It is not clear if these SNPs influence the ecology or virulence potential of these isolates. However, they do demonstrate an appreciable level of genomic diversity between strains of the same outbreak (Table 2). To further estimate the genomic diversity of this lineage, comparisons should be made to other *V. cholerae* O75 isolates from clinical and environmental isolates.

**Table 2:** ORFs with polymorphisms within the *V. cholerae* FL group.

N16961 Locus	CP1110	CP1111	CP1112	CP1113	CP1114	CP1115	CP1116	CP1117	Annotation
VC0315	A	A	A	A	G	G	A	A	CDP-diacylglycerol–serine O-phosphatidyltransferase
VC1899	C	C	C	C	T	C	C	C	hypothetical protein
VC0028	C	C	C	C	C	C	T	C	Dihydroxy-acid dehydratase
VC0031	C	C	C	C	C	C	C	A	Acetolactate synthase large subunit
VC1359	T	T	T	T	C	C	T	T	ABC-type polar amino acid transport system ATPase component
GI-26*	G	G	T	G	G	G	G	G	putative transcriptional activator ToxR
	A	A	T	A	A	A	A	A	
VCA1063	G	G	G	G	T	T	G	G	Ornithine decarboxylase

* = Not found in *V. cholerae* N16961.

### Genomic Islands, Pathogenicity Islands, and Virulence Factors

**Figure 2:**
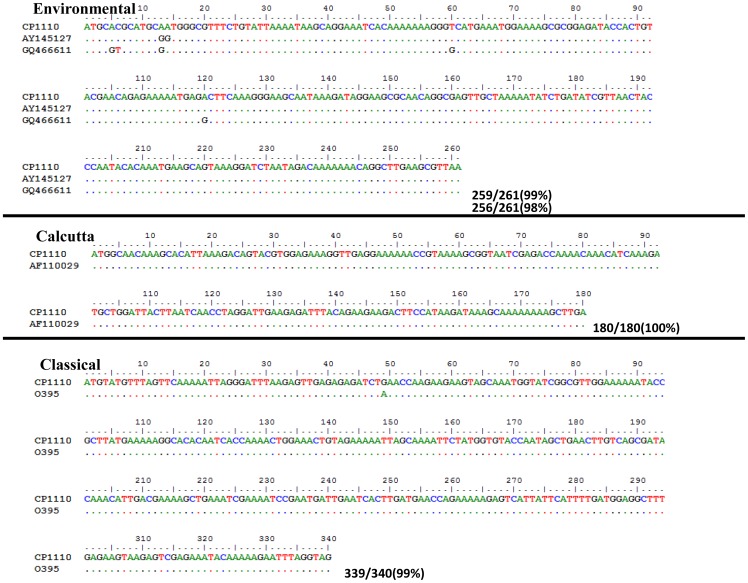
*rstR* sequences from *V. cholerae* CP1110. Sequences are aligned to their most similar homologs extracted from NCBI Genbank database. Nulceotide sequence identity is shown to the right of the last nucleotide aligned for each allele.

The *V. cholerae* FL Group isolates were determined to contain the full CTX phage encoding the cholera toxin, but the structure of this region was unresolved due to the limitations of assembly since ORFs were found on multiple contigs. For similar reasons, CTX phage copy number could not be resolved. A BLASTN analysis with *V. cholerae* N16961 and O395 as reference demonstrated the presence of regions homologous to VC1456 to VC1463 (VC0395_0512 to VC0395_0505 and VC0395_A1060 and VC0395_A1067 of *V. cholerae* O395) of the CTX phage (*ctxB*, *ctxA*, *zot*, *ace*, *orfU*,*cep*, *rstB*, *rstA*, and *rstR* ^Classical^). To infer the biotype of the cholera toxin, PCR targeting the *ctxB* gene was employed and resulted in an amplicon for primers of targeting *ctxB* ^Classical^. These PCR results are consistent with profiles of other clinically isolated *V. cholerae* strains on a global scale that suggest this cholera toxin biotype is the predominant biotype currently causing the majority of disease [34], [35]. Based on the genome sequence data, the CTX phage of the *V. cholerae* FL Group genomes were lacking the *rstR* gene of *V. cholerae* N16961 El Tor (VC1464), but did encode the *rstR* gene homologous to the one encoded in *V. cholerae* O395 Classical. To further investigate and confirm these *in silico* results, PCR targeting the *rstR* region was done and resulted in amplicons for the Calcutta, Environmental, and Classical biotypes, but not the El Tor biotype, an as-to-date uncommon combination. The *rstR* amplicons of CP1110 were subjected to Sanger sequencing and the resulting sequences were compared by BLASTN to the NCBI Genbank database for better interpretation of these results and each showed ≥99% nucleotide sequence similarity to Calcutta, Environmental, and Classical sequences (Figure 2). These amplicon sequences were compared with *V. cholerae* CP1110 reads by BLASTN to re-confirm their presence in the genome sequences. The *rstR* sequences from the *V. cholerae* FL Group were confirmed as Calcutta, Environmental, and Classical biotypes (Figure 2). The prototypical *V. cholerae* O1 El Tor strains encode *rstR* ^El Tor^ and *ctxB* ^El Tor^ while Classical strains encode *rstR* ^Classical^ and *ctxB* ^Classical^. Altered *V. cholerae* O1 El Tor strains which differ from prototypical El Tor strains in their *rstR*/*ctxB* types have recently been identified [24]. Data from this study further demonstrates the diversity of the CTX phage outside of the more frequently studied *V. cholerae* O1 strains and suggests many alleles of this phage can be associated with cholera. Cholera toxin expression was not assayed in this study.

**Figure 3:**
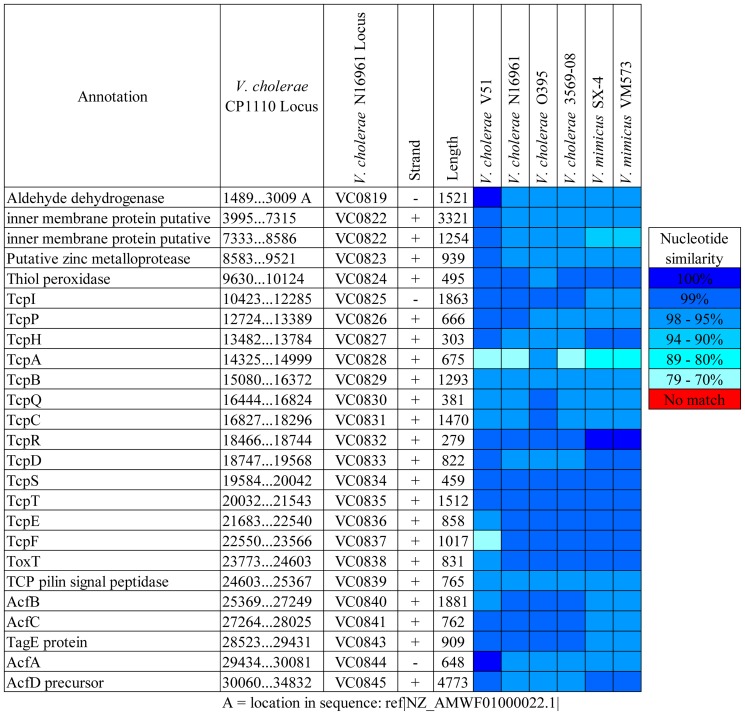
Comparative genomic analysis of *Vibrio* pathogenicity island 1 (VPI-1). VPI-1 of the *V. cholerae* FL Group is the reference sequence in a BLAST alignment with homologs of other *Vibrionaceae* genomes. Colored squares show degree of similarity.

**Figure 4:**
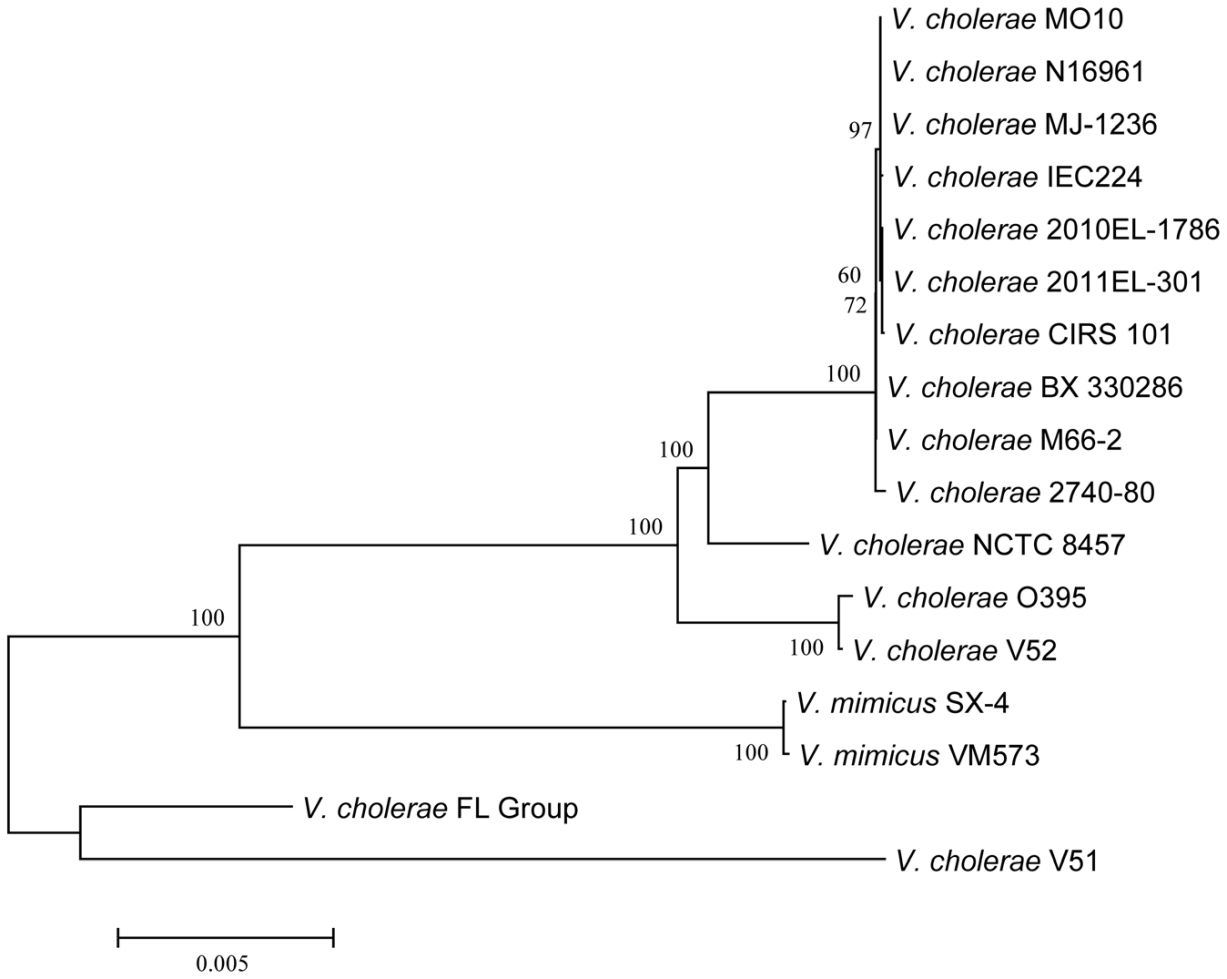
Phylogenetic analysis of *Vibrio* pathogenicity island 1 (VPI-1). Neighbor-joining tree showing evolutionary relationships of VPI-1. The calculation was based on aligned fragments of 25 orthologous genes (VC0819 to VC0845) comprising ca. 26.9 kb. Bar length = 0.005 substitutions per site.

The genomes of the eight *V. cholerae* FL Group isolates harbored *Vibrio* pathogenicity island 1 (VPI-1) encoding the toxin co-regulated pilus (TCP) shown to be responsible for biofilm formation in the intestine and a receptor for CTXΦ phage [36], [37]. VPI-1 of the *V. cholerae* FL Group is highly similar in structure to those of other clinical and environmental *V. cholerae* and *V. mimicus* (Figure 3). Interestingly, the *tcpA* gene (often used as a marker of *V. cholerae* biotype) of this group has the highest similarity with that of *V. cholerae* O395, a Classical biotype, while showing similarity of 77% with *V. cholerae* V51. However, a phylogeny of concatenated ORFs of this island demonstrates VPI-1 of the *V. cholerae* FL Group and *V. cholerae* V51 are closely related to each other from an evolutionary perspective, and significantly diverged from VPI-1 of other clinical and environmental *V. cholerae* and *V. mimicus* strains (Figure 4).

**Figure 5:**
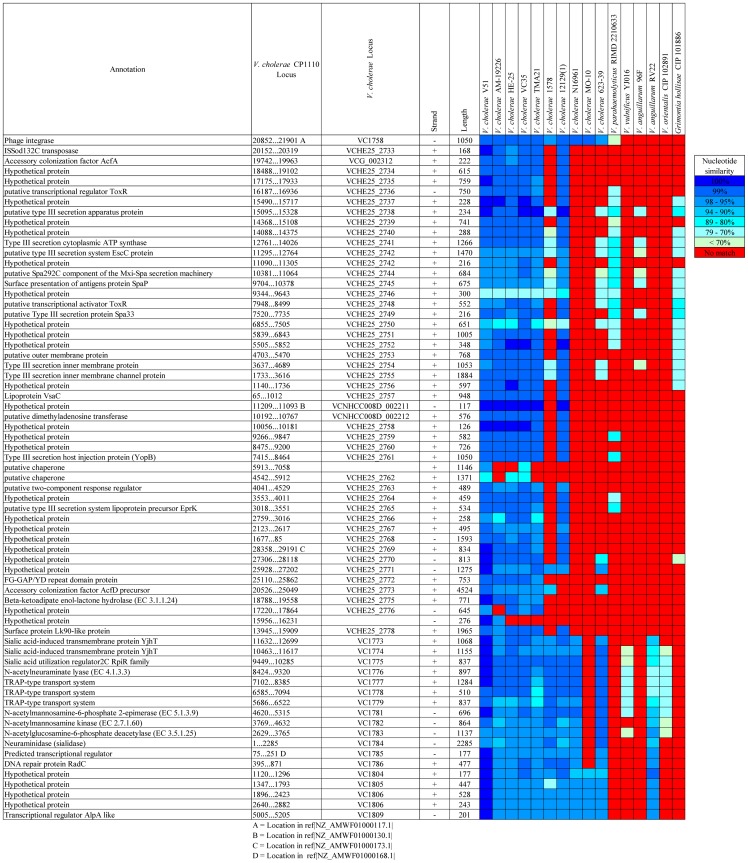
Comparative genomic analysis of *Vibrio* pathogenicity island 2 (VPI-2). VPI-2 of the *V. cholerae* FL Group is the reference sequence in a BLAST alignment with homologs of other *Vibrionaceae* genomes. Colored squares show degree of similarity.

**Figure 6:**
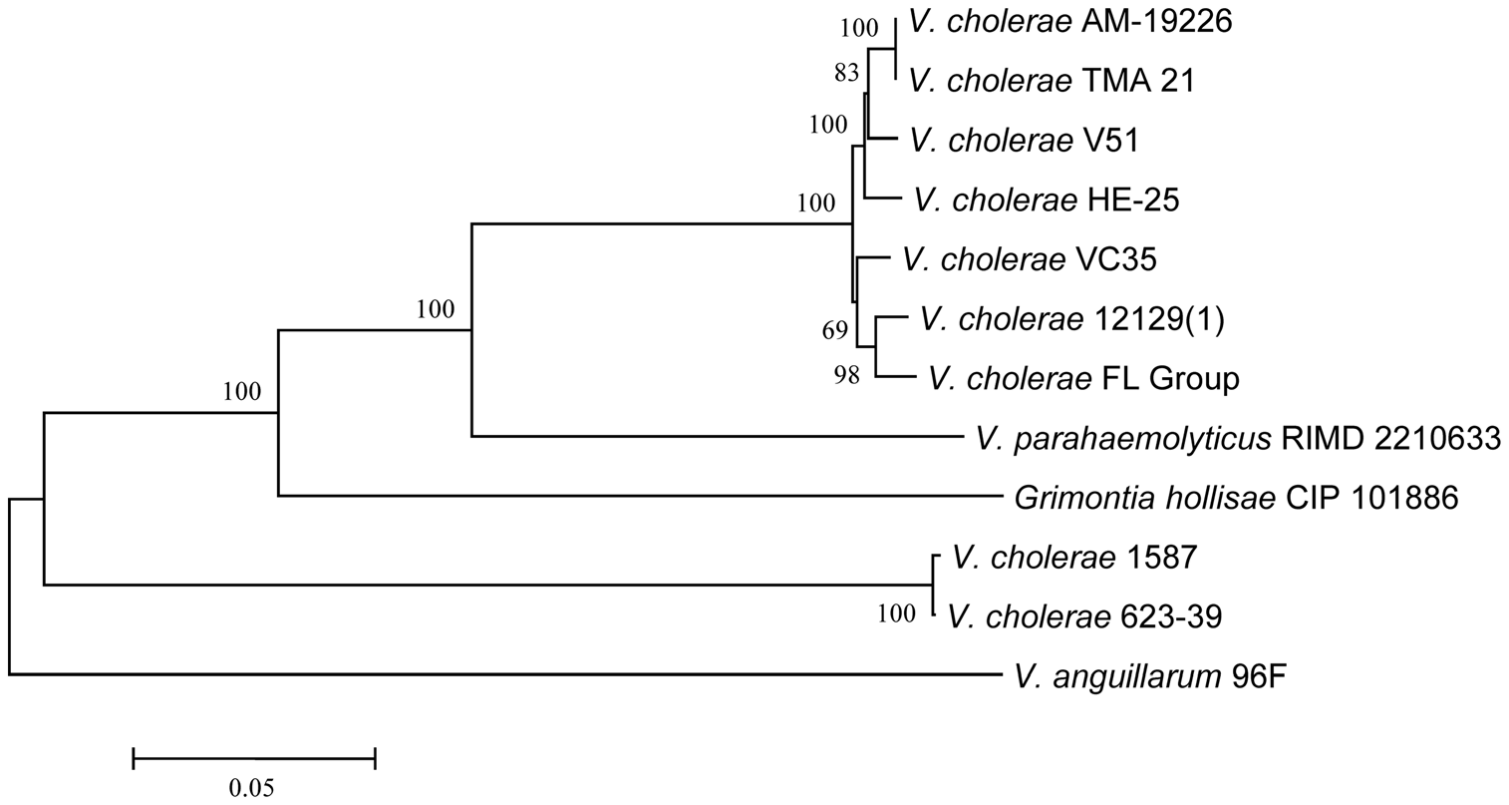
Phylogenetic analysis of ORFs conserved among all T3SS-positive *V. cholerae* and closely related species. Neighbor-joining tree inferred from an alignment of 7 orthologous genes (VCHE25_2738, VCHE25_2741, VCHE25_2742, VCHE25_2744, VCHE25_2745, VCHE25_2749, VCHE25_2754) comprising ca. 5.2 kb. Bar length = 0.05 substitutions per site.

**Figure 7:**
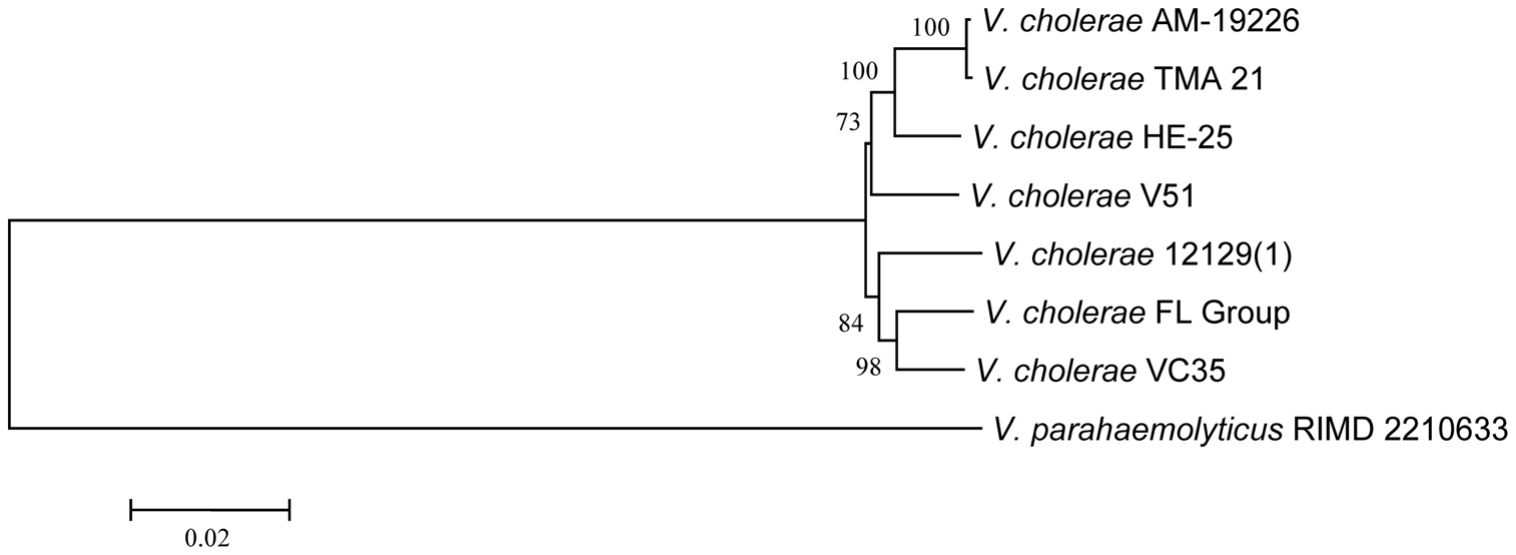
Phylogenetic analysis of most closely related T3SS. Neighbor-joining tree inferred from an alignment of 17 orthologous genes (VCHE25_2737 to VCHE25_2742, VCHE25_2745 to VCHE25_2752, and VCHE25_2754) comprising ca. 10.2 kb. Bar length = 0.02 substitutions per site.

The genomes of all *V. cholerae* FL Group isolates also encoded VPI-2, with a type III secretion system (T3SS) (Figure 5). Two divergent T3SS variants have been identified in *V. cholerae* isolates [38]. T3SS in the *V. cholerae* FL Group genomes are most similar to that of *V. cholerae* V51 and AM-19226, a non-O1 TCP-negative and CTX-negative isolate (Figure 5). The T3SS of *V. cholerae* AM-19226 has been shown to be essential for colonization of the infant rabbit intestine and associated with severe diarrhea in this model, suggesting it plays a significant role in virulence during human infections [39]. This region has been found in environmental and clinical *V. cholerae* on a global scale. For instance, *V. cholerae* HE-25, a gray water isolate from Haiti and *V. cholerae* VC35, a clinical isolate from Malaysia, both encode T3SS that is structurally and phylogenetically similar to the variant in the *V. cholerae* FL Group suggesting global distribution of this virulence factor in environmental and clinical isolates (Figure 6). A phylogeny based on conserved ORFs of this variant and of *V. parahaemolyticus* as an outgroup infers the nearest phylogenetic neighbor to T3SS in the *V. cholerae* FL Group is *V. cholerae* VC35 (Figure 7). Although this region has been shown to be part of VPI-2 variants it has been identified as a separate genomic island capable of lateral transfer between *V. cholerae* strains [12], [40].

**Figure 8:**
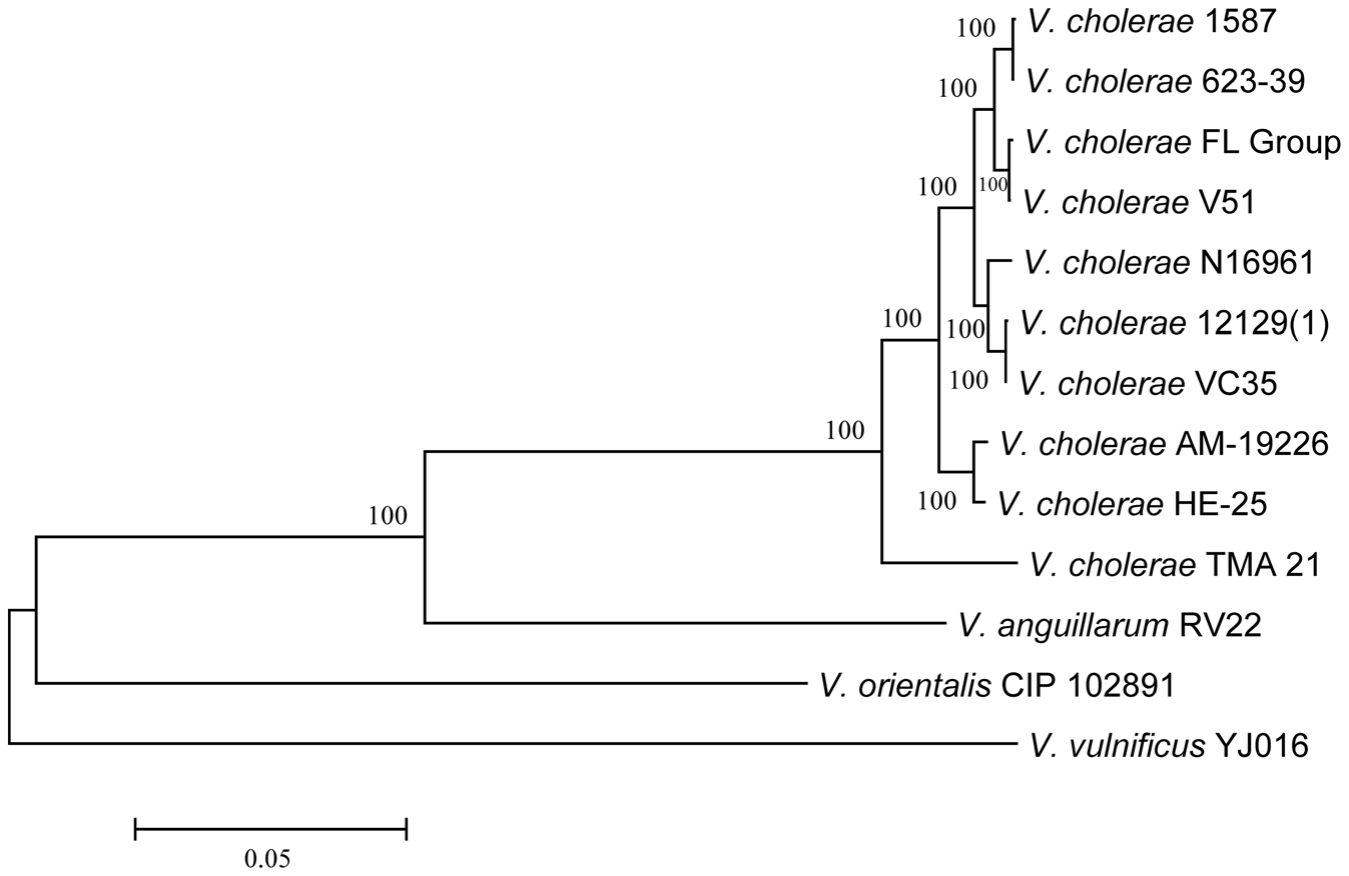
Phylogenetic analysis of sialic acid metabolism region of VPI-2. Neighbor-joining tree inferred from an alignment of 7 orthologous genes (VC1781 to VC1774) comprising ca. 5.7 kb. Bar length = 0.05 substitutions per site.

VPI-2 of the *V. cholerae* FL Group also encodes a complete sialic acid catabolism operon (homologs of VC1773 to VC1784 in the canonical VPI-2 of *V. cholerae* N16961), including a neuraminidase (sialidase) which has been shown to unmask the GM1 gangliosides of human intestinal epithelial cells, making them available to the cholera toxin [10]. A phylogeny of the sialic acid metabolism region demonstrated this operon in the *V. cholerae* FL Group is closely related to that of *V. cholerae* V51, *V. cholerae* 1587, and *V. cholerae* 623-39 (Figure 8). The phylogeny of this region is not congruent with that of the T3SS suggesting a more recent ancestral sialic acid metabolism region of the *V. cholerae* FL Group and *V. cholerae* V51 than that of the T3SS. Further, when the phylogenies of T3SS and sialic acid metabolism operons of seven *V. cholerae* strains with homologous ORFs are inferred (*V. cholerae* strains AM-19226, TMA 21, HE-25, V51, 12129(1), FL Group, and VC35), sister taxa of the *V. cholerae* FL Group T3SS remains *V. cholerae* VC35 and sister taxa of the sialic acid metabolism region of the *V. cholerae* FL Group remains *V. cholerae* V51 (data not shown). These data suggest the two regions in the *V. cholerae* FL Group originated from different sources. Morita et al. [40] demonstrated these two regions of VPI-2 could be of separate origin and the insertion locus of *V. cholerae* T3SS is exclusively in VPI-2. Interestingly, the mu-like phage region, the most variable region of the canonical VPI-2, is absent in these genomes.

**Figure 9:**
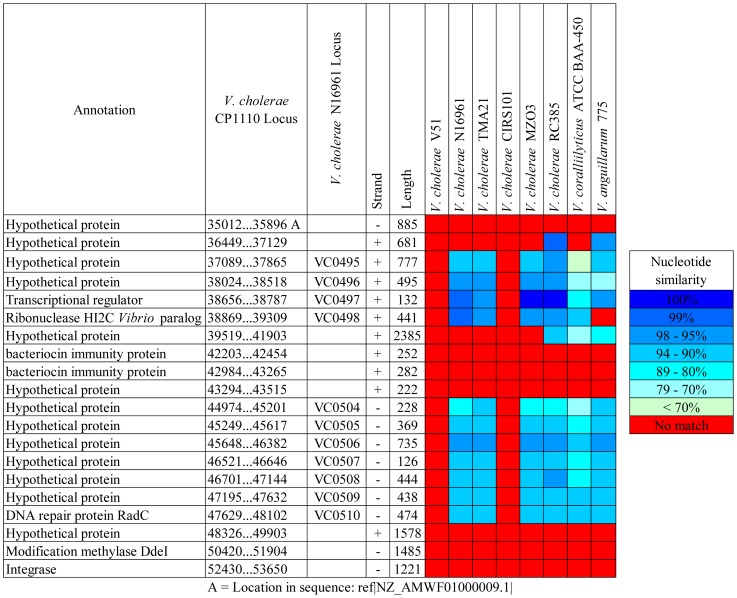
Comparative genomic analysis of *Vibrio* Seventh pandemic island II-like island. *Vibrio* Seventh pandemic II-like island of the *V. cholerae* FL Group is the reference sequence in a BLAST alignment with homologs of other *Vibrionaceae* genomes. Colored squares show degree of similarity.

**Figure 10:**
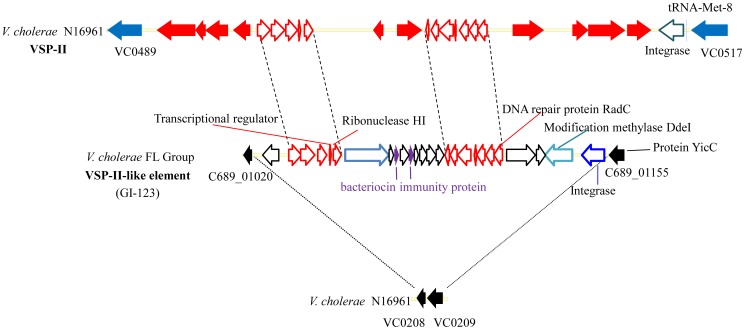
Structure of the canonical *Vibrio* Seventh Pandemic island II (VSP-II) and the VSP-II-like element of the *V. cholerae* FL Group. Structure of the canonical VSP-II element of *V. cholerae* N16961 (top), the VSP-II-like element found in the *V. cholerae* FL Group (middle) based on the annotation in NCBI Genbank, and the homologous locus of insertion of this VSP-II-like element in *V. cholerae* N16961 (bottom). Sequences homologous to VC0208 and VC0209 in *V. cholerae* V51 are found on contig NZ_DS179740 at positions 34343 to 34831 and 34897 to 35763. ORFs conserved between the two elements are outlined in red.

A VSP-II-like island was identified in the *V. cholerae* FL Group isolates with varying levels of similarity and conservation with other homologous sequences in the *Vibrionaceae* (Figure 9). This island was previously identified as GI-123, but was not well characterized [41]. Interestingly, this island does not encode the canonical integrase of VSP-II but rather one that is similar to an integrase of a not yet described genomic island in *V. cholerae* CP1033(6), a serogroup O1 strain isolated from a cholera patient in Mexico in 2000. This VSP-II-like island was not inserted at the tRNA-Met (adjacent to VC0517) where the canonical VSP-II is inserted, but rather at the locus homologous to VC0208 and VC0209, where GIs-32, 52, 68, 96, 98, 107 are inserted in other *V. cholerae* strains [12], [41]. When compared to the prototypical VSP-II island in *V. cholerae* N16961, the *V. cholerae* FL Group encodes two regions with high similarity: VC0495 to VC0498 and VC0504 to VC0510. A novel region encoding four ORFs annotated as hypothetical protein, bacteriocin immunity protein, bacteriocin immunity protein, and hypothetical protein were inserted between the two regions that are similar to the prototypical VSP-II (Figures 9 and 10). One of these hypothetical proteins comprises 794 amino acids, with cytoxic and S-type Pyocin domains, known toxins active against bacteria [42]. When compared to the NCBI nucleotide database, highest similarity is with an S-type Pyocin domain-containing protein (YP_004564713.1) of *V. anguillarum*, a marine fish pathogen. Two adjacent proteins are bacteriocin immunity proteins, with one 83 amino acids and the other 93 amino acids in length. Both have colicin immunity protein/pyocin immunity protein domains and are predicted by pSort to be in the cytoplasm of the *V. cholerae* [43]. In other species secreted pyocins are known to cause cell death among closely related strains [42]. The presence of a homologous genetic cluster in the *V. cholerae* FL Group may allow it to outcompete other *V. cholerae* strains present in the same local environment which may lead to an increased density of pyocin and pyocin immunity protein-encoding strains in a specific environment such as a single oyster bed. However, further research on pyocins in *V. cholerae* needs to be conducted to further elucidate their potential role in intra-species competition in the environment.

The VSP-II-like element in isolates of the *V. cholerae* FL Group has 12 ORFs with similarity to regions of the *V. corallilyticus* ATCC BAA-450 and *V. anguillarum* 775 genomes, with percent nucleotide identity between the ORFs ranging from 69 to 99% (Figure 9). These data suggest the suite of VSP-II-like elements is distributed not only among clinical *V. cholerae* isolates, but also environmental isolates including non-cholera vibrios. Further, the presence of similar ORFs in non-pathogenic vibrios strongly indicates a function in the natural environment.

**Figure 11:**
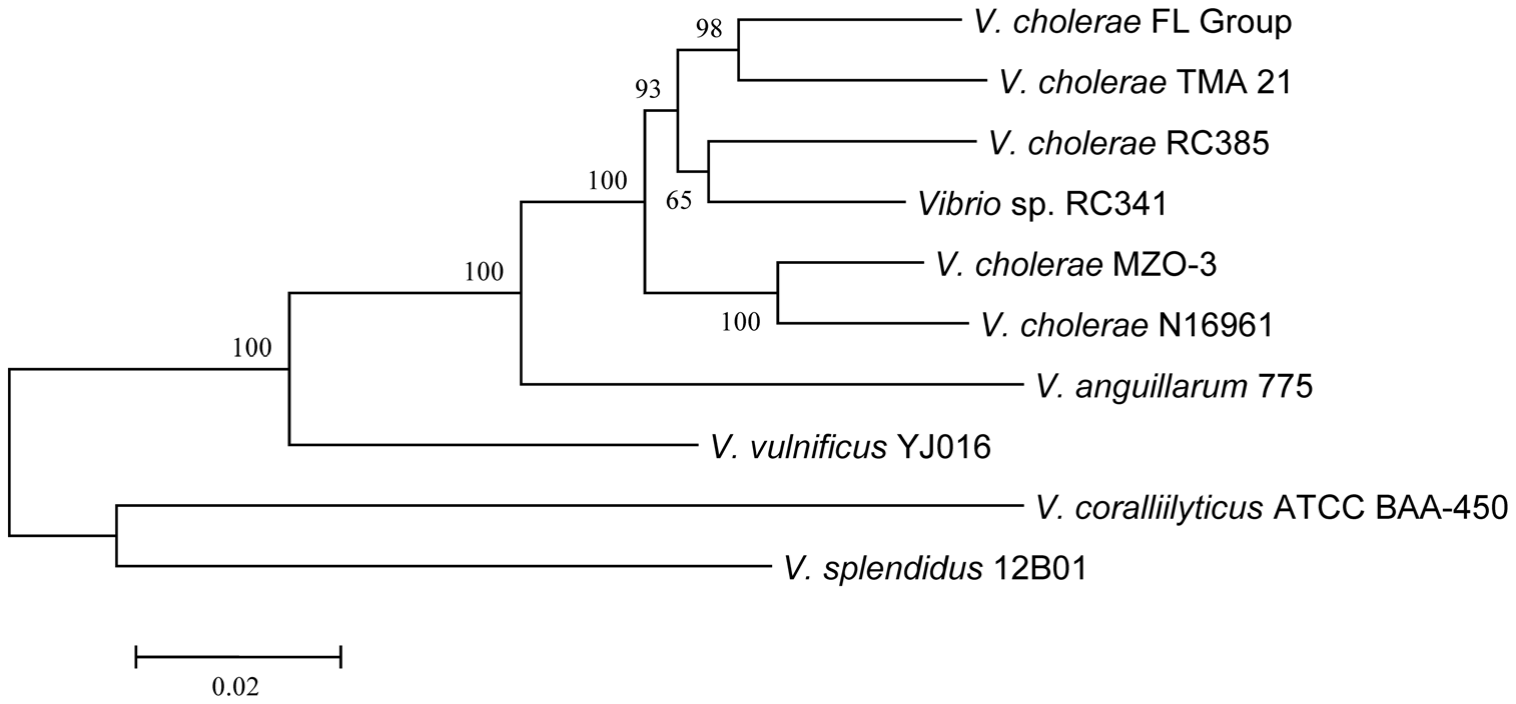
Phylogenetic analysis of *Vibrio* Seventh pandemic island II-like island. Neighbor-joining tree inferred from an alignment 8 orthologous ORFs (VC0495, VC0496, VC0504 to VC0506, VC0508 to VC0510) comprising ca. 3.7 kb. Bar represents 0.02 substitutions per site.

A phylogeny of conserved VSP-II ORFs infers these sequences of the *V. cholerae* FL Group to be closely related to *V. cholerae* TMA 21 and significantly divergent from the *V. cholerae* 7^th^ Pandemic strains (Figure 11). The subclade with which VSP-II of the *V. cholerae* FL Group clusters comprises environmental *V. cholerae* strains and *Vibrio* sp. RC341, a novel *Vibrio* species closely related to *V. cholerae* and known to cause sporadic infections in humans [15], [44], [45]. *V. cholerae* V51 does not encode this element.

**Figure 12:**
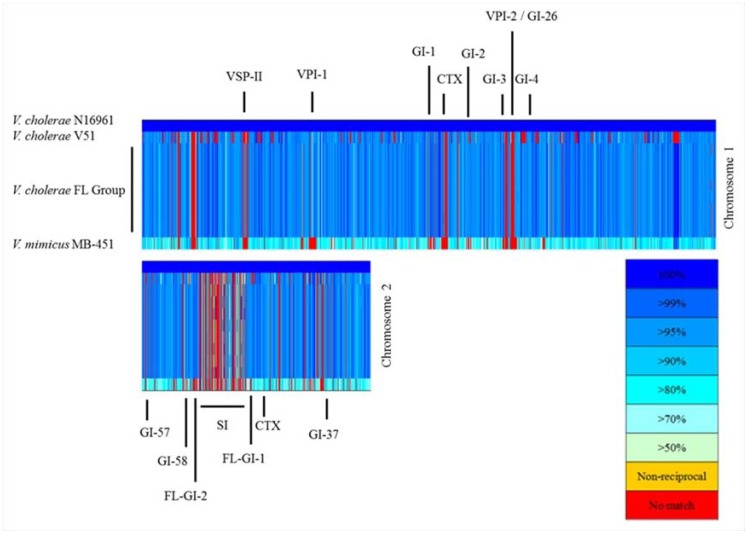
BLAST atlas of *V. cholerae* FL Group, *V. cholerae* V51, and *V. mimicus* MB-451 and *V. cholerae* N16961 genomes. *V. cholerae* N16961 is the reference genome. Genomic islands with the prefix “GI” are described by Chun et al. [12]. SI = superintegron.

**Figure 13:**
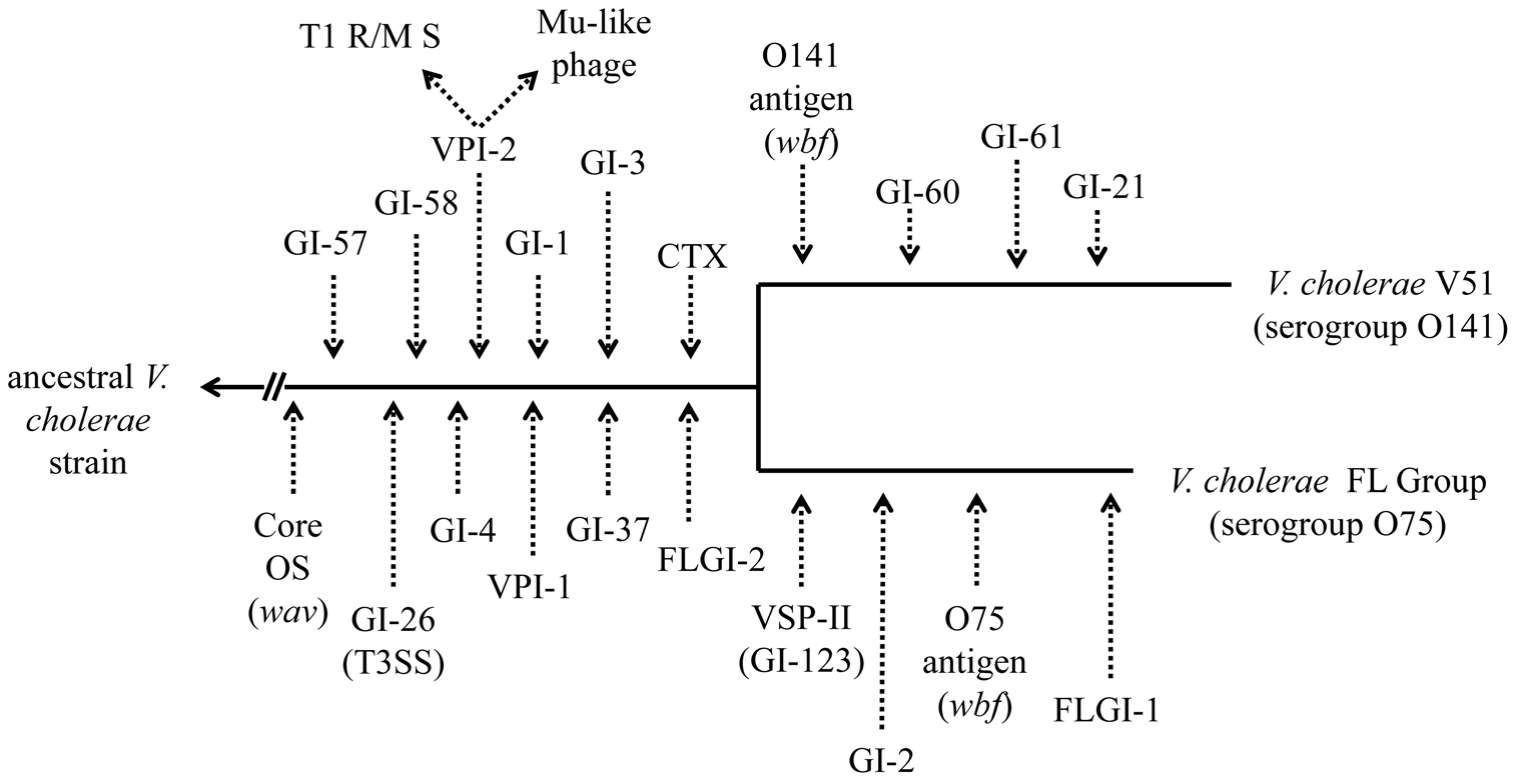
Proposed hypothetical insertions of genomic islands in the *V. cholerae* V51/*V. cholerae* FL Group clade.

The presence of genomic islands comprising the *V. cholerae* mobilome described by Chun et al. (12) was evaluated using BLASTN and BLASTP. Including VPI-1 and 2 and a VSP-II-like element, the *V. cholerae* FL Group encoded sequences with high similarity to GIs-1, 2, 3, 4, 26, 37, 57, 58, and two genomic islands not yet described and designated here as FL-GI-1 and FL-GI-2 (Figure 12). All *V. cholerae* FL Group isolates lacked VSP-I genomic island and the site of insertion does not harbor any other genomic island. Figure 13 depicts a proposed arrangement of genomic island insertion and deletion in the *V. cholerae* V51/*V. cholerae* FL Group lineage before and after the two sets of isolates (*V. cholerae* V51 and *V. cholerae* FL Group isolates) would have diverged from a common ancestor.

### Lipopolysaccharide Coding Region

**Figure 14:**
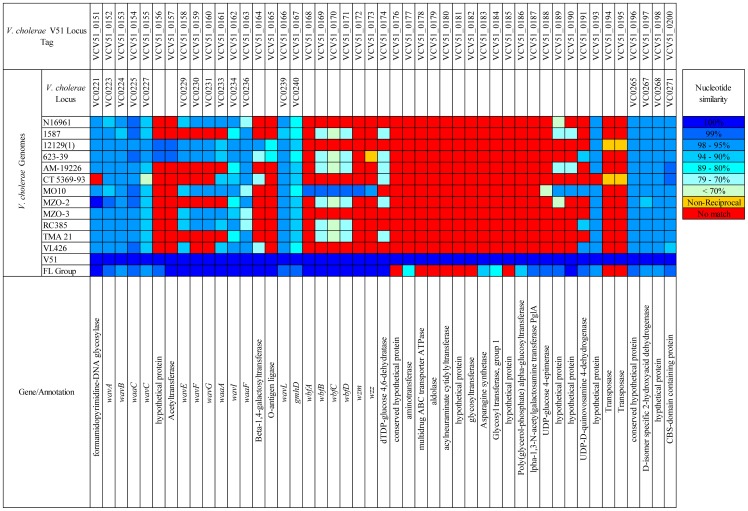
Comparative genomic analysis of LPS coding regions. Reciprocal BLAST analysis of LPS coding region with *V. cholerae* V51 as a reference.

**Figure 15:**
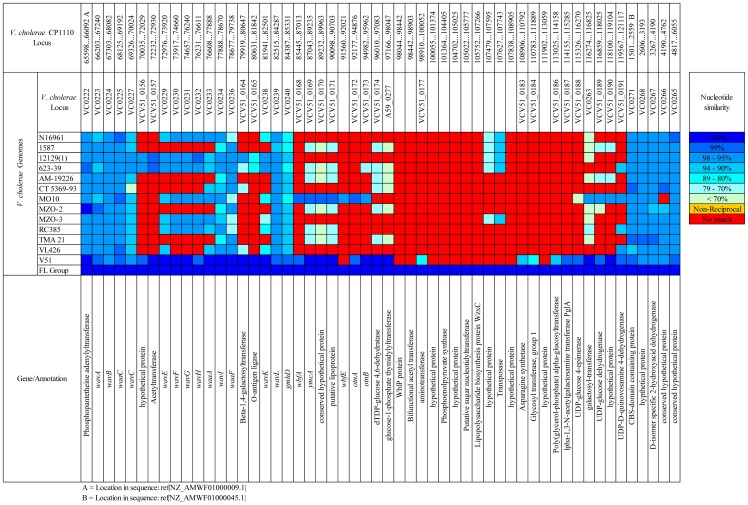
Comparative genomic analysis of LPS coding regions. Reciprocal BLAST analysis of LPS coding region with *V. cholerae* FL Group as a reference.

This region of the *V. cholerae* FL Group is ca. 60.1 kb, with the LPS core region ca. 19.1 kb and wb* region ca. 41 kb. Of all *V. cholerae* serogroup data represented in NCBI GenBank, the core oligosaccharide (OS) and the O141-antigen-specific coding regions of *V. cholerae* V51 are most similar to the homologous ORFs of *V. cholerae* FL Group (Figures 14 and 15). Of 54 identified ORFs in this region of the *V. cholerae* FL Group, *V. cholerae* V51 shares 38 with at least 95% nucleotide sequence similarity. When the O-antigen ORFs of *V. cholerae* V51 and the *V. cholerae* FL Group are compared the only observed structural differences are seven ORFs absent in the regions homologous to VCV51_0176 to VCV51_0185 in the *V. cholerae* FL Group and 11 ORFs in *V. cholerae* V51 absent in the homologous region (found between positions 98044 and 113059 in contig ref|NZ_AMWF01000009.1|). Eight ORFs were identified in the O75-antigen coding region of the *V. cholerae* FL Group isolates that have not yet been described in the O-antigen coding regions of other *V. cholerae* genomes, and these ORFs may be specific to the O75 antigen (Figures 14 and 15).

Although it is well known that this region is a hot-spot for gene transfer, it can be assumed that O141 and O75 O-antigen coding regions derived from a recent ancestral sequence based on the high level of conservation between the two, and that the difference between the two clusters arises from a substitution of ORFs specific to the O-antigen region. A similar mechanism has been suggested for the relationship between O139 and O22 serogroups [46], [47]. This substitution may have involved a ca. 18.2 kb region in the genomes of *V. cholerae* FL Group isolates and a ca. 16.2 kb region in *V. cholerae* V51 flanked by homologs found at nucleotide positions 97166 to 98047 (glucose-1-phosphate thymidylyltransferase) and 116274 to 116825 (lipid carrier:UDP-N-acetylgalactosaminyltransferase). Alternatively, three substitution events involving shorter sequences may have occurred between the flanking regions, indicated by absent ORFs (red squares in Figure 15) in reciprocal comparison. Interestingly, the serogroup with the next highest level of conservation with serogroups O141 and O75 is the epidemic-associated O139 serogroup isolate *V. cholerae* MO10.

### Phenotypic Analyses

The eight *V. cholerae* FL Group isolates were evaluated for hemolysis, motility, and proteolysis, following standard methods for testing these methods in *V. cholerae* [27]. Although not responsible for the rice water diarrhea characteristic of cholera, these virulence factors are associated with intestinal and extra-intestinal *V. cholerae* infections, as well as ecological functions in the aquatic environment [48], [49], [50], [51]. All strains are motile, proteolytic, form biofilms and are hemolytic. However, strain CP1114 demonstrated weak or incomplete hemolysis. This isolate was also weakly proteolytic, compared to the other *V. cholerae* FL Group isolates, and incomplete hemolysis may be due to incomplete processing of hemolysin by the hemagglutinin/protease [52].

**Figure 16:**
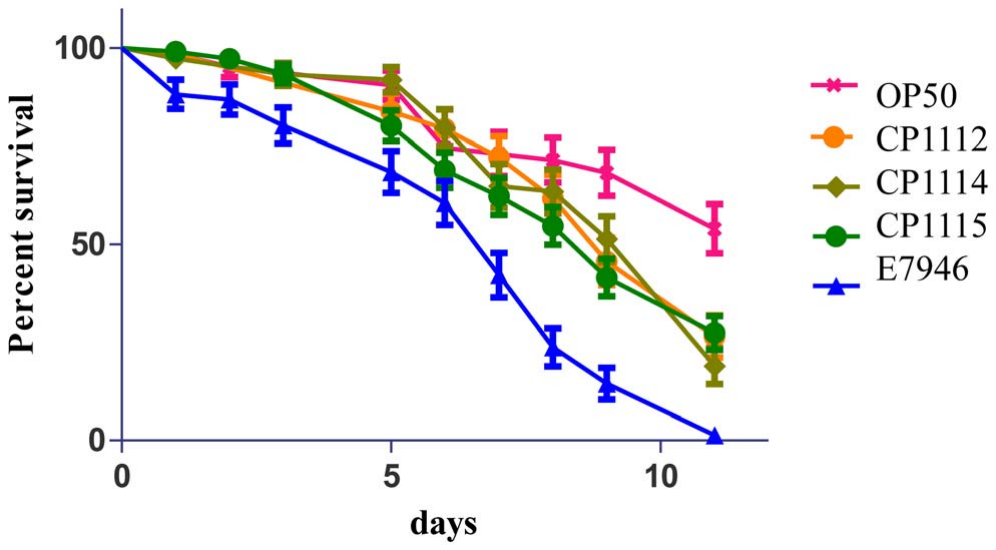
Survival curves of *C. elegans* challenged with *V. cholerae* CP1112, CP1114, CP1115, *V. cholerae* El Tor E7946, *Escherichia coli* OP50.

The *Caenorhabditis elegans* model of *V. cholerae* infection, which yields data on strength of hemolytic activity (*hlyA*) proved useful [29]. Nematodes were fed three isolates of *V. cholerae* FL Group (*V. cholerae* CP1112, 1114, and 1115). CP1115, which showed the largest zone of hemolysis on blood agar, was selected for testing. CP1114 demonstrated incomplete hemolysis and CP1112 showed a moderate zone of clearing when compared to the other isolates of the *V. cholerae* FL Group. The results demonstrated significantly more rapid lethality in nematodes fed the *V. cholerae* FL Group isolates than nematodes fed non-pathogenic *E. coli* as a control, but significantly slower lethality than nematodes fed *V. cholerae* El Tor strain E7946 (P<0.05) (Figure 16). It is concluded that all three of the *V. cholerae* FL Group isolates produced in similar *C. elegans* survival patterns. However, median survival time of worms fed isolates *V. cholerae* CP1112 and CP1115 was *ca*. nine days versus *ca*. eleven days for worms fed *V. cholerae* CP1114, the isolate with incomplete hemolysis, a consistent result based on previous observations. Interestingly, the three isolates caused a *C. elegans* die-off similar to *V. cholerae* O1 biotype Classical than to El Tor [29], not expected since *hlyA* of the *V. cholerae* FL Group does not have the same 11 bp deletion linked to the decreased hemolytic activity of *V. cholerae* O1 Classical but higher nucleotide sequence similarity with *V. cholerae* O1 El Tor N16961 than Classical O395.

Based on BiOLOG phenotypic microarray assay, all strains utilized sialic acid three to six times greater than background demonstrating a functional sialic acid catabolism operon of the VPI-2. Almagro-Moreno and Boyd [10] reported the ability to utilize sialic acid confers a competitive advantage to strains encoding this region during infection of the sialic acid-rich environment of the gut. This is due to the ability of *V. cholerae* encoding a functional sialic acid metabolism region to utilize sialic acid as a carbon source [10]. All strains also utilized maltose, which was shown by Lång et al. [53] to be related to cholera toxin and toxin co-regulated pilus production and translocation across the *V. cholerae* outer membrane. Results of the study demonstrated that a functional maltose operon is needed for virulence of *V. cholerae* [53].

The BiOLOG profiles showed similar metabolic profiles among the *V. cholerae* FL Group strains (data not shown). However, both replicates of *V. cholerae* CP1110 utilized caproic acid as carbon source while all other isolates generally did not, except isolate *V. cholerae* CP1117 which utilized this substrate in one replicate. Isolates CP1112, CP1113, and CP1116 weakly utilized caproic acid in at least one replicate. Isolate *V. cholerae* CP1115 did not utilize β-methyl-D-glucoside while the other *V. cholerae* FL Group isolates did.

## Conclusions

It is concluded the outbreak was caused by *V. cholerae* growing to a sufficiently high density in the environment (i.e., not in a single oyster) to cause multiple cases of cholera. Clonality of the isolates, including 67% of all reported cholera cases from this outbreak, demonstrates that there need not be a human vehicle of *V. cholerae* dispersal into a given geographical region prior to a cholera outbreak, as has been suggested for cholera epidemics. Further, it is concluded that genomic and phenotypic diversity exists among clinical isolates *V. cholerae* non-O1/non-O139 strains of the same outbreak, supporting a recommendation to investigate the genomics of cholera epidemics at the population level. Large-scale genomic and molecular analyses accomplished for the cholera epidemics in Haiti and Bangladesh and the recent epidemics in Nigeria and Kenya have revealed distinct *V. cholerae* populations causing disease [6], [7], [54], [55].

Because the *V. cholerae* FL Group isolates formed a monophyletic lineage with *V. cholerae* V51 serogroup O141 (a 1987 clinical isolate), we hypothesize the clade to represent a lineage of cholera-causing isolates, similar to those of the 7^th^ pandemic clade. Although, diverged from recent 7^th^ pandemic strains and older Classical and pre-7^th^ pandemic strains, from an evolutionary perspective, the virulence factors known to be involved in cholera are present in the *V. cholerae* FL Group and *V. cholerae* V51. The difference in the constellation of mobile elements and incongruent phylogenies of some elements of *V. cholerae* V51 and the *V. cholerae* FL Group suggest that, although these two groups are similar, they have independently acquired various elements from the environment, with some islands globally distributed.

Although the majority of the research on *V. cholerae* focuses on the O1 serogroup because of the major epidemics associated with these strains, *V. cholerae* non-O1/non-O139 serogroup strains should be further evaluated for contribution to the global disease burden. *V. cholerae* serogroup O141 isolates have been shown by other investigators to globally cause significant disease and many encode *ctxB* ^Classical^ [14], [30], [31], [56] as do the *V. cholerae* FL Group serogroup O75 isolates. Pathogenic *V. cholerae* causing cholera outbreaks must be characterized in a phylogenomic context and their genomic island constellations as well. It is no longer sufficient to label these *V. cholerae* strains as serogroups O1, O139, or non-O1/non-O139, without further appropriate genomic analysis.
